# Value-based care as a solution to resolve the open debate on public healthcare outsourcing in Europe: What do the available data say?

**DOI:** 10.3389/fpubh.2024.1484709

**Published:** 2024-10-22

**Authors:** Cristina Caramés, Javier Arcos, Bernadette Pfang, Ion Cristóbal, Juan Antonio Álvaro de la Parra

**Affiliations:** ^1^Quirónsalud Healthcare Network, Grupo Hospitalario Quirónsalud, Madrid, Spain; ^2^Hospital Universitario Fundación Jiménez Díaz, Madrid, Spain; ^3^Clinical and Organizational Innovations Unit, Hospital Universitario Fundación Jiménez Díaz, Madrid, Spain

**Keywords:** healthcare quality, outsourcing, value-based healthcare, patient outcomes, clinical management, patient experience

## Abstract

Controversy surrounds the current debate regarding the effects of outsourcing health services, as recent studies claim that increased outsourcing leads to reduced costs at the expense of worse patient outcomes. The goal of the value-based model is to enable healthcare systems to create more value for patients, and evidence points to improvements in public health outcomes, patient experience, and health expenditure in systems incorporating components of value-based healthcare. Some emerging evidence indicates promising results for outsourced hospitals which follow a value-based model of healthcare delivery. Although additional future studies are still needed to confirm these benefits, value-based healthcare merits discussion as a new perspective on the public versus private management debate. In fact, we argue that outsourcing to value-based health providers could represent a valid alternative for public health management, encouraging greater competition within the healthcare sector while ensuring quality of care for both public and private sectors.

## Introduction

European health systems currently fall under one of three co-existing management models: National Health Services, Social Insurance-based Systems or Mixed Model systems. These models differ regarding aspects such as financing, economic policies, and service delivery. In European countries with established National Health Services (United Kingdom, Spain, Italy, Portugal, and Denmark, among others), universal access to health care is guaranteed and financed through public taxation. A strong primary care network is at the core of National Health Services, with general practitioners acting as first port of call for the patients in their catchment area and as gatekeepers to specialist care. In this model, hospitals and other care facilities are owned by the state, but management may be delegated to non-profit or for-profit private organizations (1). Social Insurance-based systems rely on compulsory contributions from employers and employees to insurance funds used to reimburse healthcare providers. Healthcare providers can be either public or private (2). Examples of European countries following this model of healthcare include Germany, France, and the Netherlands (3–5), all of which provide universal access to healthcare. Finally, mixed systems, such as that of Bulgaria (6), involve a significant amount of private funding from voluntary insurance schemes or out-of-pocket reimbursement.

Over the last decade, there has been an evident shift in Europe toward outsourcing the management of publicly to private organizations, usually on the grounds of controlling national healthcare expenditure and increasing competitivity in the healthcare sector. However, while reversing this trend seems unlikely, largescale outsourcing has sparked concern that the financial interests of private organizations are undermining quality of care, leading to a heated debate in which defenders of healthcare outsourcing argue that increasing competition will improve healthcare service performance, while opponents contend that, although outsourcing seems to be an unavoidable consequence of financial pressure and increased demand for healthcare, it will surely lead to worse patient outcomes (7). As the Mixed model of healthcare is relatively uncommon in Europe, this article focuses mainly on potential effects of outsourcing in National Health Systems and Social Insurance-based models. While funding sources differ between models, both systems rely in lesser or greater measure on publicly owned entities to deliver care. In the National Health System blueprint, all resources are owned and managed by the state; Social Insurance-based models such as that of Germany own and manage around a third of national hospitals (8, 9). Strong national regulation of healthcare in both NHS and SI models means that healthcare is generally accessible to all residents. Both types of systems have increasingly turned to outsourcing publicly owned facilities and services since the early 2000s, citing similar reasons for this strategy: existence of financial constraints; need to improve system efficiency; need to incorporate new technologies; and the search for improved quality of care (7). Outsourcing can be performed on different scales, from contracting uncritical services such as laundry or catering, to outsourcing the management of medical facilities, to contracting highly critical services such as cardiology or intensive care (10, 11). Possible risks to data security, potential negative effects on the quality of care offered to patients, and cost-motivated staff reductions are concerns voiced by opponents of outsourcing in both systems (12).

This article aims to make a constructive contribution to the current “public versus private” healthcare debate by adding a new perspective which is currently gaining importance in countries with universal access to healthcare: value-based healthcare delivery. Although VBHC was conceived in the United States as a form of driving competition between healthcare providers, in Europe, where most countries grant universal access to care, VBHC is seen as driving cooperation between organizations to ensure patient-centered, high-quality care while reducing related costs. At the heart of VBHC is the objective of providing value to patients (patient-centered care) at the lowest possible cost, an objective that transcends ownership or management status (13). Thus, elements of VBHC have been successfully implemented in different models of healthcare, and in both privately and publicly managed facilities. Several successful case studies in VBHC in Europe include the private, non-profit Diabeter Network in the Netherlands, which provides integrated, person-centered care for people with Type 1 diabetes and has demonstrated improved clinical outcomes while reducing expenditure (14); the privately-owned Martini Klinik in Germany, which is an internationally recognized center for the treatment of prostate cancer (15); the Quirónsalud network in Spain, which manages both private and public hospitals (16–21); and the public-private Netherlands Heart Network (22, 23). In this article, we highlight legitimate concerns about outsourcing healthcare and summarize available evidence on outcomes in outsourced care, underlining the importance of taking different forms of care delivery into account when analyzing healthcare services and patient outcomes. We review the concept of value-based care and present recent examples of implementation of this model of care delivery. Finally, we summarize current evidence suggesting that value-based care may improve clinical outcomes and overall quality of care, indicating that *how* healthcare is managed may be even more important than *who* manages it.

## Potential effects of outsourcing in countries with universal access to healthcare: an update

Several studies have evaluated the impact of healthcare outsourcing in countries with universal access to healthcare, using different perspectives and parameters (Table 1).

**Table 1:** Summary of cited studies on the effects of outsourcing healthcare services in high-income countries.

Author	Year	Country	Study type	Comparison	Study outcome	Results
Beckman et al. (30)	2020	Sweden	Retrospective observational study	Period following increased privatization of healthcare in Sweden.	Health utilization	Increased privatization improved access to primary care overall, although this improvement was more pronounced in patients with above-median income.
Goodair and Reeves (26)	2024	8 different high-income countries (including USA)	Systematic review	Public sector healthcare facilities with a component of outsourced care	Aggregated effects of outsourcing	Outsourcing care can be associated with negative consequences (increases in avoidable hospital admissions, higher treatable mortality, staffing reductions).
Goodair and Reeves (27)	2022	UK	Retrospective, observational study	Clinical commissioning groups with higher percentage of outsourcing compared to those with less outsourcing	Treatable mortality rates	Treatable mortality rates were associated with clinical commissioning groups with higher percentages of outsourcing
Gustafsson et al. (31)	2024	Sweden	Interrupted time series	Preintervention and postintervention periods defined as years before and after the establishment of privately owned primary health providers.	Inequities in avoidable hospitalizations	Reduction in overall rates of avoidable hospitalizations and reduction in age disparities. Higher socioeconomic inequities in avoidable hospitalizations.
Hebrang et al. (32)	2003	Croatia	Structured interviews with primary care physicians	Privatized hospitals and non-privatized primary care practicies	Accessibility to care	Privatized practices demonstrated better accessibility to primary care.
Kruse et al. (24)	2018	Europe	Systematic review	Public hospitals and private hospitals	Efficiency, accessibility, quality of care	In most cases, higher efficiency in public hospitals. Accessibility in private sector associated with higher socio-economic levels. No significant differences in quality of care.
Mosquera et al. (29)	2021	Sweden	Interrupted time series	Preintervention and postintervention periods defined as years before and after the establishment of privately owned primary health providers.	Hospitalizations and emergency department visits for ambulatory care sensitive conditions	Counties with higher rates of privatization displayed less favorable results regarding emergency department visits for ambulatory care sensitive conditions
Ribera et al. (28)	2006	Spain	Prospective, observational study	Three publicly managed hospitals and two outsourced hospitals	Risk-adjusted hospital mortality of coronary surgery	Mortality rates were slightly lower for privately managed centers after adjusting for baseline patient characteristics.

A systematic review comparing European public and private hospitals in terms of efficiency, accessibility, and quality of care (24) concluded that, with some exceptions, public hospitals were more efficient than their private counterparts. In this review, accessibility to private sector was associated with higher social-economic levels. Evidence for differences in quality of care between public and private hospitals was inconclusive. The review included studies published before June 2017 from countries with different healthcare management models. Individual studies used different indicators to measure efficiency, accessibility, and quality of care, making comparison between public and private hospitals difficult, and limiting the generalizability of these findings. This systematic review provides valuable information about the state of European public and private healthcare sectors before the COVID-19 pandemic.

A qualitative analysis of 24 different studies performed in the last decade examining the perspectives of both public and private social care providers (such as long-term geriatric and mental health facilities) highlighted that under austere financial conditions – such as ongoing budget cuts in the healthcare sector – it is a mistake to assume that trying to increase competency of public providers through outsourcing will improve quality in healthcare delivery (25). Notably, both public and private providers expressed similar issues with contracts, commenting that unstable regulation and insufficient prices led to deteriorated employment conditions and service quality in both sectors. A limitation of this study is that it focused exclusively on social care providers, without including the perspective of acute or primary care.

A recent publication by Goodair and Reeves (26) investigated the aggregated effects of outsourcing in high-income countries, including studies from 2009 to 2022. Notably, this systematic review highlighted that outsourcing care can cause negative consequences due to the development of cost reduction strategies leading to reductions in staffing, increases in avoidable hospital admissions as reported by one study, and higher treatable mortality as observed in three studies. The authors conclude that outsourcing may indeed reduce costs, but potentially at the expense of quality of care. However, it is important to consider that only 13 studies from eight different healthcare systems were included in the analysis, less than half of which focused on health outcomes. A small sample size, heterogeneities among included healthcare systems, and lack of evidence from the majority of European countries entail that these conclusions must be taken cautiously.

In a previous work, the same authors performed an observational study analyzing the effects of outsourcing healthcare on treatable mortality rates between 2013 and 2020 in England (27). Interestingly, they found that the increase in the percentage of outsourcing was significantly associated with an increase in treatable mortality, hypothesizing that this finding was caused by lower quality of the healthcare services offered by for-profit providers. As in the previous case, this study presents some key limitations, such as the lack of data on the complexity of the population attended or information on the specific services provided by the supplier. In fact, the authors stated that a causal relationship cannot be established between increased mortality and the outsourcing of health care. Their findings contrast with a study from Spain, in which patients undergoing coronary bypass surgery in outsourced hospitals presented significantly lower mortality rates than in publicly managed hospitals, after adjusting for surgical risk and comorbidities (28).

A series of studies focusing on the effects of increased outsourcing of primary care in Sweden found that overall higher levels of outsourcing were associated with lower rates of avoidable hospital admissions across the country (although with higher rates of avoidable admissions in regions with more outsourced care), as well as with increased overall access to primary care and lower disparities in access to care across age groups, although with higher rates of emergency care use and disparities in hospitalizations and access to care across socioeconomic groups (29–31). The effects of increased outsourcing in primary care were also examined in a study from the Croatian healthcare system, which found that outsourcing was associated with an increase in access to care (32).

Altogether, bearing in mind the complexity of healthcare scenario and the increasing prevalence of outsourcing in Europe, we believe that policymakers should analyze different models of healthcare delivery in privately managed care in order to determine which strategies can effectively increase quality of care.

## Value-based care delivery as a novel optimal strategy for healthcare management

At the end of the 20th century, a revolutionary new concept – evidence-based medicine – emphasized the need for clinical decisions to be based on the best available scientific evidence along with the best clinical expertise (33). Moreover, decisions should integrate patient’s preferences to ensure the highest possible quality of life at lower costs (34). In 2006, emergence of value-based healthcare in the United States of America marked a milestone in the evolution of evidence-based medicine as a form of healthcare delivery.

The value-based model of healthcare delivery challenges the traditional model of healthcare by incorporating changes geared toward improving outcomes that truly matter to patients, and in the most sustainable way (13, 35). Redesigning health systems to focus on value for patients involves not only measuring clinician reported outcomes (CROMs), but also incorporating patient-reported outcomes and experience measures (PROMs and PREMs). In the value-based model of care delivery, capitation-based payment is exchanged for pay-for-performance which aligns reimbursement with value to incentivize “quality of care” over “quantity of care.” Value-based care promotes the organization of care around medical conditions through integrated practice units, thus promoting truly patient-centered care, as well as harnessing information technology, optimizing the geography of care, and ensuring systems integration to improve equity and sustainability in access to healthcare by streamlining the logistics of care delivery.

When analyzing the effect of value-based healthcare, it is important to consider that the potential applications of this model could differ across healthcare systems. However, the objective of value-based care – to enable healthcare systems to create more value for patients – remains the same (36). Various studies have examined the effectiveness of the different components of value-based healthcare on quality of care. The importance of measuring patient-reported outcomes is indisputable, and many studies have demonstrated that patients participating in PROMs initiatives from a range of clinical specialties present better outcomes (including improved symptom control, quality of life, and patient-provider communication) than non-participants (37–39). Evidence for pay-for-performance comes mostly from US and UK studies which show heterogeneous effects on quality of care, with results varying widely among centers (40, 41). Several studies suggest that pay-for-performance is associated with better clinical outcomes including immunization rates and control of risk factors for cardiovascular disease (42–44). A recent study from France demonstrated overall improvement in public health outcomes for primary care centers participating in a pay-for-performance program, including higher rates of chronic disease follow-up and cancer prevention, as well as lower rates of iatrogenesis and inappropriate antibiotic use (45). Healthcare-related expenditure also improved over the three-year study period. The implementation of integrated practice units has been associated with decreased complications of treatment and hospital readmission rates (19, 46), increased access to care even for uninsured patients (47, 48), improved patient experience (19), and reductions in healthcare costs. Finally, few studies focus on the other components of value-based care (systems integration, optimizing geography of care through national centers of excellence to provide care for exceedingly complex patients, and developing a robust information technology platform) and more research is necessary to confirm the effect of these components on quality of care and clinical outcomes (49).

Although many European countries are embracing VBHC as a solution to the current state of healthcare, many obstacles exist. One of the greatest challenges to VBHC is that current healthcare systems have been around for many decades and cannot be redesigned “from scratch”; in fact, healthcare provider and payer resistance is one of the greatest barriers to VBHC implementation. The initial costs of implementing VBHC – for example, expenditure on enabling IT platforms – can be problematic, especially in underfunded countries. However, in the long run, studies point to cost savings due to improved patient outcomes and care-related costs (50). While the original concept of VBHC includes six components which should be implemented simultaneously, most successful real-life implementations of VBHC in Europe focus on one or two components, such as measuring costs and outcomes for every patient, or organizing care into integrated practice units. Many studies cite legal barriers and adherence to traditional, “pay for service” payment models as a major challenge toward implementing VBHC, as healthcare providers are not incentivized to provide higher value care and multiple obstacles exist impeding reorganization of care (51). The concept of “bundled payment” and “pay for performance,” too, have its potential drawbacks. In systems that are highly dependent on private insurance without strong government regulation, such as the USA, patients with risk factors are prone to being “lemon dropped,” while in systems with a majority of publicly owned facilities and tighter government regulation (such as most European countries), hospitals in socially deprived areas or with a greater complexity case-mix could be unjustly penalized for worse outcomes (52). One of the pillars of VBHC – measuring costs and outcomes for every patient – can be burdensome to both patients and healthcare providers, pointing to the need to implement strategies to reduce survey fatigue and increased paperwork, especially in understaffed healthcare settings (53, 54). The organization of care into Integrated Practice Units, while often applicable in the USA setting, is often deemed unpractical in the European setting (55); however, advocates of VBHC have proposed developing standardized patient pathways as an alternative to IPUs to achieve better, patient-centered organization of care (56).

## Does VBHC transcend the public-private debate by improving healthcare outcomes independently of hospital ownership/management status?

Current indicators used to compare public and private healthcare outcomes include clinical outcomes (such as mortality rates and hospital-acquired infection), efficiency metrics (such as average length of hospital stay and surgical waiting lists), patient satisfaction metrics, and cost analyses. To determine if VBHC can transcend the public-private debate by improving these outcomes independently of hospital ownership/management status, we performed a scoping review of the literature, focusing on studies published since 2020 in European countries. We included only those studies describing already-implemented VBHC solutions, excluding theoretical or pilot studies. Included studies followed a quantitative research design, excluding mixed-methods and qualitative approaches. Included endpoints were clinical outcomes (including patient-reported outcomes), efficiency metrics, patient satisfaction outcomes and/or cost analysis.

We used the following query to search MedLine via PubMed: (“Value-Based Health Insurance”[MeSH Terms] OR “value-based care”[Text Word] OR “value-based healthcare”[Text Word] OR “value-based health care”[Text Word] OR “valuebased care”[Text Word] OR “value-based care”[Text Word] OR “value-based healthcare”[Text Word] OR “value-based health care”[Text Word] OR “VBHC”[Text Word]) AND (2020:2024[pdat]).

Our query returned 1770 results. After screening 176 articles, we finally included 11 studies were included in the review. The results of our review are presented in Supplementary Table S1. According to our findings, the initial, quantitative evidence on VBHC in Europe demonstrates that adopting a value-based attitude to healthcare and implementing components of VBHC can, in fact, improve outcomes in both public and private settings, Interestingly, two of the 11 examples included large-scale initiatives in which both public and private providers participated, highlighting the potential of VBHC to drive value-based collaboration in European healthcare. Also, the studies focusing on VBHC in private centers demonstrated improvements not only in efficiency metrics such as overall length of stay (57) and increased productivity (57, 58), but also increased quality of life for patients (58), improved clinical outcomes (59), and improved perception of quality of care (60), underlining the potential of VBHC to ensure quality of care and patient-centeredness in outsourced and privatized services.

This literature review has several limitations. Firstly, the published studies may be subject to selection bias. The search query did not include specific search terms for each of the components of VBHC, and so we may have excluded some studies focusing on individual components of the value-based framework. Secondly, we only included studies during the last 5 years as the healthcare landscape has changed significantly since the COVID-19 pandemic. However, this approach may have excluded several reports. Finally, although we differentiated between VBHC in private and public healthcare, we did not identify any studies comparing outcomes of VBHC between public and outsourced services. Although our findings point to VBHC as a potential solution to the theoretical drawbacks of outsourcing, further research is necessary to confirm this hypothesis.

## Discussion

This article summarizes several studies which analyze effects derived from healthcare outsourcing. A shared limitation is that these studies fail to mention the potential impact of alternative models of health-care delivery, such as value-based healthcare, on quality of care when choosing to outsource. This issue is probably caused by lack of available data at the time of publication. However, an increasing amount of evidence on the positive effects of value-based care on private and public healthcare has been published in recent years, and this data should be analyzed and considered as a new perspective on the current “public versus private” debate.

It has been claimed that, for private companies, reducing costs is easier than improving quality of care, but recent data suggest that implementing value-based healthcare leads to improved clinical outcomes, better patient experience, and more sustainable care – regardless of management type. Kaiser Permanente, a major US private health system with a value-based model of care delivery, has demonstrated better patient outcomes and lower use of resources than German Social Insurance hospitals operating on a traditional care model (61). Among countries with National Health Services, official data from the Regional Outcomes Observatory of Madrid (Spain) (62) show that four publicly owned hospitals outsourced to a value-based, for-profit healthcare provider and covering a population of close to 1 million patients present lower-than-average inpatient mortality and medical and surgical complications, as well as lower surgical backlogs and higher patient experience scores than their publicly managed counterparts.

Irrespectively of differing characteristics of national health systems regarding funding, payer and provider status, European healthcare is highly regulated by state and local government. Thus, government authorities have an important role in facilitating VBHC in privatized settings, for example, by designing contracting policies not only based on cost-effectiveness but also on clinical and patient-reported outcomes. Bundled payment or pay-for-performance can also incentivize private providers to improve quality of care. A typical argument against bundled payment in the privatized setting is the risk of “lemon dropping,” that is, avoiding patients whose social or health profile could influence outcomes negatively. However, in European healthcare, this scenario is highly mitigated by government regulation and medical ethics. In fact, yearly audit data from outsourced hospitals in Spain shows a higher-complexity case mix than publicly managed counterparts (62), while data from Germany show that public and privately run hospitals make similar losses from caring for uninsured patients (63).

While the use of PROMs and PREMs to motivate clinical decisions can give patients a voice in healthcare delivery, some concerns have been voiced regarding potential disparities. Socially disadvantaged groups are often less health-literate and may not participate equally in PROMs and PREMs programs (64). Breast cancer researchers observed that non-responders in a PROM program were more frequently of older age, non-English speaking, and non-white, pointing to the need for addressing the digital divide and language barriers in patient-reported data collection programs (65). Although risk selection could theoretically hamper the potential of VBHC (66), emerging evidence suggests that participation in value-based payment models reduces race and economic disparities (67, 68). Further studies are necessary to confirm the effects of VBHC on healthcare disparities in European countries.

In our opinion, European healthcare policy makers should bear in mind that the “public versus private” debate is not the only factor to consider when trying to solve the current discussion about which is the best healthcare system for our society. In fact, one of the advantages of the private sector is that changes occur faster, allowing new initiatives to be tested in a limited environment before being adopted by the public sector. In this sense, initial evidence from Europe points to the effectiveness of the value-based model of healthcare delivery (34). Meanwhile, preliminary data shows that outsourcing to providers with a value-based strategy may have advantages both in terms of profitability, health outcomes and patient experience compared to the traditional model and may represent an optimal form of contracting.

In conclusion, further research is necessary to fully elucidate the effects of different models of health-care delivery on quality of care when transitioning from public to private management. Furthermore, the progressive adoption of emerging health policies in European countries to encourage “value-based” decision-making represents an essential strategy that must be analyzed within a constructive debate on the impact and consequences of health-care outsourcing. In the meantime, we agree that “quantity over quality” is not an option when health-care outsourcing is concerned.

## Data Availability

The original contributions presented in the study are included in the article/Supplementary material, further inquiries can be directed to the corresponding authors.

## References

[1] Gaeta M Campanella F Capasso L Schifino GM Gentile L Banfi G. An overview of different health indicators used in the European health systems. J Prev Med Hyg. (2017) 58:E114–20. PMID: 28900351 PMC5584080

[2] Tulchinsky TH. Bismarck and the long road to universal health coverage. Case Stud Public Health. (2018) 131:8. doi: 10.1016/B978-0-12-804571-8.00031-7

[3] Birault F Mignot S Caunes N Boutin P Bouquet E Pérault-Pochat MC. The characteristics of care provided to population(s) in precarious situations in 2015. A preliminary study on the universal health cover in France. Int J Environ Res Public Health. (2020) 17:3305. doi: 10.3390/ijerph17093305 32397452 PMC7246706

[4] Busse R Blümel M Knieps F Bärnighausen T. Statutory health insurance in Germany: a health system shaped by 135 years of solidarity, self-governance, and competition. Lancet. (2017) 390:882–97. doi: 10.1016/S0140-6736(17)31280-1 28684025

[5] Kuipers T van de Pas R Krumeich A. Is the healthcare provision in the Netherlands compliant with universal health coverage based on the right to health? A narrative literature review. Glob Health. (2022) 18:1–11. doi: 10.1186/s12992-022-00831-7 PMC8976435 35366916

[6] Dimova A Rohova M Moutafova E Atanasova E Koeva S Panteli D. Bulgaria health system review. Health Syst Transit. (2012) 14:1–186. PMID: 22894828

[7] Goodair B. ‘Accident and emergency?’ Exploring the reasons for increased privatisation in England’s NHS. Health Policy. (2023) 138:104941. doi: 10.1016/J.HEALTHPOL.2023.104941, PMID: 37979466 PMC10933725

[8] Tiemann O Schreyögg J. Effects of ownership on hospital efficiency in Germany. Bus Res. (2009) 2:115–45. doi: 10.1007/BF03342707

[9] Klenk T. Ownership change and the rise of a for-profit hospital industry in Germany. Policy Stud. (2011) 32:263–75. doi: 10.1080/01442872.2011.561694

[10] Lorence DP Spink A. Healthcare information systems outsourcing. Int J Inf Manag. (2004) 24:131–45. doi: 10.1016/j.ijinfomgt.2003.12.011

[11] Roberts J Henderson J Olive L Obaka D. A review of outsourcing of services in health care organizations. J Outsourc Organizat Informat Manag. (2013):1–10. doi: 10.5171/2013.985197

[12] Augurzky B Scheuer M. Outsourcing in the German hospital sector. Serv Ind J. (2007) 27:263–77. doi: 10.1080/02642060701207080

[13] Porter ME Lee TH. The strategy that will fix healthcare. Harv Bus Rev. (2013) 91:24.24730167

[14] Steinbeck V Fresemann ML Busse R. Value-based healthcare as an opportunity for patient management at the individual and system level. Inn Med (Heidelb) (2024). 890–8. 10.1007/s00108-024-01767-3 39093324

[15] Würnschimmel C Maurer T Knipper S von Breunig F Zoellner C Thederan I. Martini-Klinik experience of prostate cancer surgery during the early phase of the COVID-19 pandemic. BJU Int. (2020) 126:252–5. doi: 10.1111/bju.15115, PMID: 32424990 PMC7276763

[16] del Olmo M Córdoba R Gómez-Meana Á Herrero A Pascual A Cabello Á. Implementing a broad digital framework to drive network strategy through PROMs and PREMs. NEJM Catal Innov Care Deliv. (2023) 4:83. doi: 10.1056/CAT.23.0083

[17] Short Apellaniz J Álvaro de la Parra JA Gomez-Meana A Carabias L Cordoba R Arcos J. Leveraging telemedicine to reduce ED overcrowding: the Quirónsalud virtual urgent care program. NEJM Catal Innov Care Deliv. (2023) 4:422. doi: 10.1056/CAT.22.0422

[18] Álvaro de la Parra JA del Olmo Rodríguez M Caramés Sánchez C Blanco Á Pfang B Mayoralas-Alises S. Effect of an algorithm for automatic placing of standardised test order sets on low-value appointments and attendance rates at four Spanish teaching hospitals: an interrupted time series analysis. BMJ Open. (2024) 14:e081158. doi: 10.1136/bmjopen-2023-081158, PMID: 38267242 PMC10824031

[19] del Olmo M Córdoba R Gómez-Meana Á Herrero A Pascual A Cabello A. The HOPE project: improving Cancer patient experience and clinical outcomes through an integrated practice unit and digital transformation. NEJM Catal. (2023) 4:414. doi: 10.1056/CAT.22.0414

[20] Flores-Balado Á Castresana Méndez C Herrero González A Mesón Gutierrez R de las Casas Cámara G Vila Cordero B. Using artificial intelligence to reduce orthopedic surgical site infection surveillance workload: Algorithm design, validation, and implementation in 4 Spanish hospitals. Am. J. Infect. Control. (2023) 51:1225–1229. doi: 10.1016/J.AJIC.2023.04.165 37100291

[21] Guadalajara H Lopez-Fernandez O León Arellano M Domínguez-Prieto V Caramés C Garcia-Olmo D. The Role of Shared Decision-Making in Personalised Medicine: Opening the Debate. Pharmac. (2022) 15:215. doi: 10.3390/PH15020215 PMC8880233 35215327

[22] Theunissen L Cremers HP Dekker L Janssen H Burg M Huijbers E. Implementing value-based health care principles in the full cycle of care: the pragmatic evolution of the Netherlands heart network. Circ Cardiovasc Qual Outcomes. (2023) 16:E009054. doi: 10.1161/CIRCOUTCOMES.122.009054 36924224

[23] Wyffels E Beles M Baeyens A Croeckaert K de Potter T van Camp G. Same day discharge strategy by default in a tertiary catheterization Laboratory in Belgium: value based healthcare-change in practice. Health Policy. (2023) 132:104826. doi: 10.1016/J.HEALTHPOL.2023.104826 37087953

[24] Kruse FM Stadhouders NW Adang EM Groenewoud S Jeurissen PPT. Do private hospitals outperform public hospitals regarding efficiency, accessibility, and quality of care in the European Union? A literature review. Int J Health Plann Manag. (2018) 33:e434–53. doi: 10.1002/hpm.2502, PMID: 29498430 PMC6033142

[25] Bach-Mortensen AM Barlow J. Outsourced austerity or improved services? A systematic review and thematic synthesis of the experiences of social care providers and commissioners in quasi-markets. Soc Sci Med. (2021) 276:113844. doi: 10.1016/J.SOCSCIMED.2021.113844, PMID: 33773477

[26] Goodair B Reeves A. The effect of health-care privatisation on the quality of care. Lancet Public Health. (2024) 9:e199–206. doi: 10.1016/S2468-2667(24)00003-3, PMID: 38429019

[27] Goodair B Reeves A. Outsourcing health-care services to the private sector and treatable mortality rates in England, 2013-20: an observational study of NHS privatisation. Lancet Public Health. (2022) 7:e638–46. doi: 10.1016/S2468-2667(22)00133-5, PMID: 35779546 PMC10932752

[28] Ribera A Ferreira-González I Cascant P Pons JMV Permanyer-Miralda G. Evaluation of risk-adjusted hospital mortality after coronary artery bypass graft surgery in the Catalan public healthcare system. Influence of hospital management type (ARCA study). Rev Esp Cardiol. (2006) 59:431–40. doi: 10.1016/S1885-5857(06)60791-3 16750140

[29] Mosquera PA San Sebastian M Burström B Hurtig AK Gustafsson PE. Performing through privatization: an ecological natural experiment of the impact of the Swedish free choice reform on ambulatory care sensitive conditions. Front Public Health. (2021) 9:4998. doi: 10.3389/FPUBH.2021.504998 PMC8200664 34136446

[30] Beckman A Anell A. Changes in health care utilisation following a reform involving choice and privatisation in Swedish primary care: a five-year follow-up of GP-visits. BMC Health Serv Res. (2013) 13:452. doi: 10.1186/1472-6963-13-452, PMID: 24171894 PMC4228471

[31] Gustafsson PE Fonseca-Rodríguez O Castel Feced S San Sebastián M Bastos JL Mosquera PA. A novel application of interrupted time series analysis to identify the impact of a primary health care reform on intersectional inequities in avoidable hospitalizations in the adult Swedish population. Soc Sci Med. (2024) 343:116589. doi: 10.1016/J.SOCSCIMED.2024.116589, PMID: 38237285

[32] Hebrang A Henigsberg N Erdeljic V Foro S Vidjak V Grga A. Privatization in the health care system of Croatia: effects on general practice accessibility. Health Policy Plan. (2003) 18:421–8. doi: 10.1093/heapol/czg050, PMID: 14654518

[33] Sackett DL Rosenberg WMC. The need for evidence-based medicine. J R Soc Med. (1995) 88:330–4. 8544145 10.1177/014107689508801105 PMC1295384

[34] Sackett DL Rosenberg WMC Gray JAM Haynes RB Richardson WS. Evidence based medicine: what it is and what it isn’t. BMJ. (1996) 312:71–2. doi: 10.1136/bmj.312.7023.71 8555924 PMC2349778

[35] Marzorati C Pravettoni G. Value as the key concept in the health care system: how it has influenced medical practice and clinical decision-making processes. J Multidiscip Healthc. (2017) 10:101–6. doi: 10.2147/JMDH.S122383, PMID: 28356752 PMC5367583

[36] Teisberg E Wallace S O’Hara S. Defining and implementing value-based health care: a strategic framework. Acad Med. (2020) 95:682–5. doi: 10.1097/ACM.0000000000003122, PMID: 31833857 PMC7185050

[37] Bele S Chugh A Mohamed B Teela L Haverman L Santana MJ. Patient-reported outcome measures in routine pediatric clinical care: a systematic review. Front Pediatr. (2020) 8:364. doi: 10.3389/FPED.2020.00364, PMID: 32850521 PMC7399166

[38] Graupner C Kimman ML Mul S Slok AHM Claessens D Kleijnen J. Patient outcomes, patient experiences and process indicators associated with the routine use of patient-reported outcome measures (PROMs) in cancer care: a systematic review. Support Care Cancer. (2021) 29:573–93. doi: 10.1007/s00520-020-05695-4, PMID: 32875373 PMC7767901

[39] Steinbeck V Langenberger B Schöner L Wittich L Klauser W Mayer M. Electronic patient-reported outcome monitoring to improve quality of life after joint replacement: secondary analysis of a randomized clinical trial. JAMA Netw Open. (2023) 6:E2331301. doi: 10.1001/jamanetworkopen.2023.31301, PMID: 37656459 PMC10474554

[40] Wagenschieber E Blunck D. Impact of reimbursement systems on patient care – a systematic review of systematic reviews. Health Econ Rev. (2024) 14:22. doi: 10.1186/S13561-024-00487-6 38492098 PMC10944612

[41] Slawomirski L Hensher M Campbell J deGraaff B. Pay-for-performance and patient safety in acute care: a systematic review. Health Policy. (2024) 143:105051. doi: 10.1016/J.HEALTHPOL.2024.105051, PMID: 38547664

[42] Huang J Yin S Lin Y Jiang Q He Y du L. Impact of pay-for-performance on management of diabetes: a systematic review. J Evid Based Med. (2013) 6:173–84. doi: 10.1111/jebm.12052, PMID: 24325374

[43] Jia L Meng Q Scott A Yuan B Zhang L. Payment methods for healthcare providers working in outpatient healthcare settings. Cochrane Database Syst Rev. (2021) 2021:CD011865. doi: 10.1002/14651858.CD011865.PUB2, PMID: 33469932 PMC8094987

[44] Langdown C Peckham S. The use of financial incentives to help improve health outcomes: is the quality and outcomes framework fit for purpose? A systematic review. J Public Health (Oxf). (2014) 36:251–8. doi: 10.1093/pubmed/fdt077, PMID: 23929885

[45] Sanchez MA Sanchez S Bouazzi L Peillard L Ohl-Hurtaud A Quantin C. Does the implementation of pay-for-performance indicators improve the quality of healthcare? First results in France. Front. Public Health. (2023) 11:3806. doi: 10.3389/FPUBH.2023.1063806 PMC10035788 36969635

[46] Low LL Tan SY Ng MJM Tay WY Ng LB Balasubramaniam K. Applying the integrated practice unit concept to a modified virtual Ward model of Care for Patients at highest risk of readmission: a randomized controlled trial. PLoS One. (2017) 12:e0168757. doi: 10.1371/JOURNAL.PONE.0168757, PMID: 28045940 PMC5207403

[47] Borde D Agana-Norman DFG Leverence R Photos L Shuster J Lukose K. Outcomes of an integrated practice unit for vulnerable emergency department patients. BMC Health Serv Res. (2023) 23:1449. doi: 10.1186/S12913-023-10067-9, PMID: 38129783 PMC10740262

[48] Williams DV Liu TC Zywiel MG Hoff MK Ward L Bozic KJ. Impact of an integrated practice unit on the value of musculoskeletal care for uninsured and underinsured patients. Healthc. (2019) 7:16–20. doi: 10.1016/j.hjdsi.2018.10.001, PMID: 30391168

[49] van Staalduinen DJ van den Bekerom P Groeneveld S Kidanemariam M Stiggelbout AM van den Akker-van Marle ME. The implementation of value-based healthcare: a scoping review. BMC Health Serv Res. (2022) 22:270. doi: 10.1186/S12913-022-07489-2, PMID: 35227279 PMC8886826

[50] Islam MK Ruths S Jansen K Falck R Mölken MRV Askildsen JE. Evaluating an integrated care pathway for frail elderly patients in Norway using multi-criteria decision analysis. BMC Health Serv Res. (2021) 21:884. doi: 10.1186/S12913-021-06805-6, PMID: 34454494 PMC8400755

[51] Bandurska E Ciećko W Olszewska-Karaban M Damps-Konstańska I Szalewska D Janowiak P. Value-based integrated care (VBIC) concept implementation in a real-world setting-problem-based analysis of barriers and challenges. Healthcare. (2023) 11:1110. doi: 10.3390/HEALTHCARE11081110 37107944 PMC10138009

[52] Bohler F Garden A Brock C Bohler L. Value-based healthcare payment models: a wolf in sheep’s clothing for patients and clinicians. Ann Med. (2024). 56:2948. doi: 10.1080/07853890.2024.2382948 PMC11271071 39046804

[53] Amini M Oemrawsingh A Verweij LM Lingsma HF Hazelzet JA Eijkenaar F. Facilitators and barriers for implementing patient-reported outcome measures in clinical care: an academic center’s initial experience. Health Policy. (2021) 125:1247–55. doi: 10.1016/j.healthpol.2021.07.001, PMID: 34311981

[54] Nguyen H Butow P Dhillon H Sundaresan P. A review of the barriers to using patient-reported outcomes (PROs) and patient-reported outcome measures (PROMs) in routine cancer care. J Med Radiat Sci. (2021) 68:186–95. doi: 10.1002/jmrs.421, PMID: 32815314 PMC8168064

[55] Lewis S. Value-based healthcare: is it the way forward? Future Healthc J. (2022) 9:211. doi: 10.7861/fhj.2022-0099 36561818 PMC9761467

[56] Cossio-Gil Y Omara M Watson C Casey J Chakhunashvili A Gutiérrez-San Miguel M. The roadmap for implementing value-based healthcare in European university hospitals—consensus report and recommendations. Value Health. (2022) 25:1148–56. doi: 10.1016/j.jval.2021.11.1355, PMID: 35779941

[57] Cecconi M Goretti G Pradella A Meroni P Pisarra M Torzilli G. Value-based preoperative assessment in a large academic hospital. J Anesth Analg Crit Care. (2024) 4:42. doi: 10.1186/S44158-024-00161-7, PMID: 38978057 PMC11232329

[58] Goretti G Marinari GM Vanni E Ferrari C. Value-based healthcare and enhanced recovery after surgery implementation in a high-volume bariatric Center in Italy. Obes Surg. (2020) 30:2519–27. doi: 10.1007/s11695-020-04464-w, PMID: 32096016 PMC7260281

[59] Van Dam VS Van Zijl FVWJ Kremer B Datema FR. The rhinoplasty healthcare monitor: an update on the practical and clinical benefits after 10 years of prospective outcome measurements. Facial Plast Surg. (2023) 40:539–45. doi: 10.1055/a-2218-7189 38016662

[60] Van Veghel D Soliman-Hamad M Schulz DN Cost B Simmers TA Dekker LRC. Improving clinical outcomes and patient satisfaction among patients with coronary artery disease: an example of enhancing regional integration between a cardiac Centre and a referring hospital. BMC Health Serv Res. (2020) 20:1–8. doi: 10.1186/s12913-020-05352-w PMC7268761 32493361

[61] Simon B Navarro R Reddy NC Convissar JL Rabrenovich V Paxton E. Patient pathway comparison for Total hip replacement in the United States and Germany — why the payment model matters. NEJM Catal. (2023) 4:456. doi: 10.1056/CAT.22.0456

[62] Observatorio de resultados del Servicio Madrileño de Salud|Comunidad de Madrid. Available at: https://www.comunidad.madrid/servicios/salud/observatorio-resultados-servicio-madrileno-salud (accessed May 17, 2024).

[63] Zimmer M. Refusal of patients: care for people without health insurance in German emergency departments. BMC Med Ethics. (2024) 25:56. doi: 10.1186/S12910-024-01059-3, PMID: 38755596 PMC11097448

[64] Shapiro LM Katz P Stern BZ Kamal RN. Equitable integration of patient-reported outcomes into clinical practice – opportunities, challenges, and a roadmap for implementation. J Am Acad Orthop Surg. (2024) 32:187–95. doi: 10.5435/JAAOS-D-23-00798, PMID: 38194644

[65] Srour MK Tadros AB Sevilimedu V Nelson JA Cracchiolo JR McCready TM. Who are we missing: does engagement in patient-reported outcome measures for breast Cancer vary by age, race, or disease stage? Ann Surg Oncol. (2022) 29:7964–73. doi: 10.1245/s10434-022-12477-1, PMID: 36149608 PMC10328095

[66] Leao DLL Pavlova M Groot W. Risk selection reduces efficiency of value-based healthcare. Int J Health Plann Manag. (2023) 38:1088–96. doi: 10.1002/hpm.3648, PMID: 37665086

[67] Kilaru AS Liao JM Wang E Zhao Y Zhu J Ng G. Association between mandatory bundled payments and changes in socioeconomic disparities for joint replacement outcomes. Health Serv Res. (2024) 59:4369. doi: 10.1111/1475-6773.14369 PMC11366957 39128893

[68] Canterberry M Rastegar JS Haq S Sylwestrzak G Boudreau E. Race disparities in emergency department utilization: analyzing the role of value-based payment among Medicare advantage beneficiaries. Healthc. (2024) 12:100748. doi: 10.1016/J.HJDSI.2024.100748, PMID: 38943723

