# Supplementary material for: Value-based care as a solution to resolve the open debate on public healthcare outsourcing in Europe: What do the available data say?
Source: Front Public Health. 2024 Oct 22;12:1484709. doi: 10.3389/fpubh.2024.1484709 (PMC11539035; doi:10.3389/fpubh.2024.1484709)
Supplement: Supplementary file 1 [file Table_1.DOCX]

Supplementary Table 1: Quantitative studies on the effects of implementing Value-Based healthcare (VHBC) in Europe, 2019-2024

| Author | Year | Country | Ownership/management status | Elements of VBHC conceptualized in the study | Conceptualization of VBHC | Initiative | Components of VBHC implemented | Implementation strategies | Reported effects |
| --- | --- | --- | --- | --- | --- | --- | --- | --- | --- |
| Ahluwalia et al.(1) | 2021 | United Kingdom | Publicly owned and managed hospital | N/A | Not specified | Day surgery unit (DSU) pathway  Time driven activity-based costing | Organize care into integrated practice units (IPUs)  Measuring costs and outcomes for every patient | Not specified | Lower waiting times  Lower overall length of stay  Increased financial benefits  Increased patient satisfaction |
| Bandurska et al. (2) | 2023 | Poland | Multicenter, public-administration coordinated initiative | Concept | “Relation between patient-centered outcome measures and the  cost of achieving improvement in these results.” | Coordinated specialist care for patients after myocardial infarction with implementation of patient pathways | Integrate care delivery across separate facilities  Measuring costs and outcomes for every patient | Not specified | Lower mortality rates  Decreased waiting time for cardiac rehabilitation  Increased patient satisfaction |
| Cecconi et al.(3) | 2024 | Italy | Privately owned and managed hospital | Concept  Goals | “An innovative framework for transforming healthcare systems, along with a strategic agenda for moving to a high-value healthcare delivery system.  The objective of VBHC is to establish the value that matters to patients as opposed to expanding service volume.” | Revision of a preoperative pathway across different units of a large surgical hospital, to align it with the principles of VBHC | Organize care into integrated practice units (IPUs)  Integrate care delivery across separate facilities | Multidisciplinary meetings  Improve guideline adherence | Reduced preoperative testing  Lower overall length of stay |
| Fernández-Salido et al.(4) | 2024 | Spain | Seven publicly owned, publicly managed primary care centres | Value  Goals | “(VBHC aims for) the delivery of value to patients with the understanding that value is defined as improved health outcomes achieved from the entire care process.” | Implementation of a comprehensive  and personalized approach based on motivational interviewing on the reduction of frailty in older adults, supported by a digital tool (ValueCare project) | Measuring costs and outcomes for every patient  Build an enabling information technology platform | Motivational  social prescription sessions  Implementation of monthly social workshops in the participating primary  care centers  Monitoring and support of the intervention through the ValueCare  digital solution | Increased physical health  Reduced use of healthcare resources |
| Gabriel et al. (5) | 2019 | United Kingdom | Publicly owned, publicly managed hospital | Value | “Value in healthcare is defined as outcomes that matter to patients and carers relative to the costs of delivering those outcomes and is measured over the complete care pathway.” | Care pathway | Organize care into integrated practice units (IPUs)  Measuring costs and outcomes for every patient | Not specified | No significant differences in clinical outcomes  Increased value of care  Decreased pathway costs |
| Goretti et al. (6) | 2020 | Italy | Privately owned and managed hospital | Concept | “VBHC is a strategic management framework that maximizes the ratio between health outcomes and costs. Developed in 2006, it is based on three acting principles: (i) building value for the patients; (ii) basing the organization of medical practice on medical conditions  and care cycles; (iii) measuring outcomes and economic costs.” | Organizational pathway | Organize care into integrated practice units (IPUs)  Measuring costs and outcomes for every patient | Multi-professional team meetings (including patients)  Establish patient engagement | Increased productivity  Improved quality of life |
| Islam et al.(7) | 2021 | Norway | Publicly owned and managed home-care and short-term nursing home facilities | N/A | Not defined | Integrated care program for frail elderly patients | Measuring costs and outcomes for every patient  Integrate care delivery across separate facilities. |  | Increased primary care costs  Decreased hospital stay and nursing home care -related costs  Participants scored lower on feeling autonomous  Participants scored higher on enjoyment of life |
| Montesinos Gálvez et al. (8) | 2020 | Spain | Twelve publicly owned, publicly managed hospitals | Concept | “VBHC is a new sanitary paradigm, born from technological advancements, scientific innovation, and changes in patient health culture. It involves a break away from traditional medicine, based on the quantity of services provided, to one based on the value of these services.” | Organizational innovation model based on Advanced Practice Nursing with Ostomies (APN-O) | Organize care into integrated practice units (IPUs) | Not specified | Increase in cost of 136.99 euros/patient  Increase in Quality-Adjusted Life Years |
| Nielsen et al. (9) | 2023 | Denmark | Publicly owned, publicly managed | Goals | “(VBHC) focuses on maximizing the value of care for patients by improving the quality of care and gives emphasis on preventive measures, which help in cutting down future costs.” | Effects of pay-for-performance in psoriasis patients | Measuring costs and outcomes for every patient  Pay-for-performance | Not specified | Improved clinical outcomes (PASI (Psoriasis Area and Severity Index) and Patient Benefit Index) |
| Theunissen et al.(10) | 2023 | Netherlands | A broad initiative including pubic and private entities from primary, secondary and tertiary care | Value | “Optimal patient-relevant outcomes divided by the lowest possible costs.” | Platform to enable primary, secondary and tertiary cardiac care prioritize improving patient value, using data from daily practice to continuously measure and improve the added value of healthcare delivery | Measuring costs and outcomes for every patient  Integrate care delivery across separate facilities | Incorporation of program manager and methodological expert  Multidisciplinary network teams around most prevalent heart conditions  Steering group including representatives of primary care, hospital physicians and hospital management | Improvement in blood pressure levels  Reductions in  Hospitalizations  Improvement in event free survival |
| Van Dam et al.(11) | 2024 | Netherlands | Privately owned and managed hospital | N/A | Not defined | Effects of implementing a PROMs program (rhinoplasty health care monitor) | Measuring costs and outcomes for every patient | Implementation of a custom web-based dashboard | Improved clinical outcomes after rhinoplasty (NOSE scale sum score and functional VAS score) |
| Van Veghel et al. (11) | 2020 | Netherlands | Two privately owned, privately managed hospitals (one large and one small hospital) | Concept | “VBHC is a promising strategy to increase patient value” | Regional integration of healthcare delivery systems and different interventions to improve clinical outcomes and patient satisfaction | Measuring costs and outcomes for every patient  Integrate care delivery across separate facilities | Daily discussion sessions  Multidisciplinary meetings  Include consultant resources.  Improve guideline adherence | Improved overall patient satisfaction and perception of quality of care. |

1. Ahluwalia R, Cook J, Raheman F, Karuppaiah K, Colegate-Stone T, Tavakkolizadeh A, Kavarthapu V, Sinha J. Improving the efficiency of ankle fracture care through home care and day-surgery units: Delivering safe surgery on a value-based healthcare model. *The Surgeon* (2021) 19:e95–e102. doi: 10.1016/J.SURGE.2020.08.004

2. Bandurska E, Ciećko W, Olszewska-Karaban M, Damps-Konstańska I, Szalewska D, Janowiak P, Jassem E. Value-Based Integrated Care (VBIC) Concept Implementation in a Real-World Setting-Problem-Based Analysis of Barriers and Challenges. *Healthcare (Basel)* (2023) 11: doi: 10.3390/HEALTHCARE11081110

3. Cecconi M, Goretti G, Pradella A, Meroni P, Pisarra M, Torzilli G, Montorsi M, Antonino S, Zerbi A, Castoro C, et al. Value-based preoperative assessment in a large academic hospital. *Journal of Anesthesia, Analgesia and Critical Care* (2024) 4:1–10. doi: 10.1186/S44158-024-00161-7/TABLES/3

4. Fernández-Salido M, Alhambra-Borrás T, Garcés-Ferrer J. Efficacy of a Comprehensive and Personalised Approach for Frail Older People in Valencia (Spain): A Pre-Post Controlled Trial. *Healthcare (Basel)* (2024) 12: doi: 10.3390/HEALTHCARE12171754

5. Gabriel L, Casey J, Gee M, Palmer C, Sinha J, Moxham J, Colegate-Stone TJ. Value-based healthcare analysis of joint replacement surgery for patients with primary hip osteoarthritis. *BMJ Open Qual* (2019) 8:549. doi: 10.1136/bmjoq-2018-000549

6. Goretti G, Marinari GM, Vanni E, Ferrari C. Value-Based Healthcare and Enhanced Recovery After Surgery Implementation in a High-Volume Bariatric Center in Italy. *Obes Surg* (2020) 30:2519. doi: 10.1007/S11695-020-04464-W

7. Islam MK, Ruths S, Jansen K, Falck R, Mölken MR van, Askildsen JE. Evaluating an integrated care pathway for frail elderly patients in Norway using multi-criteria decision analysis. *BMC Health Serv Res* (2021) 21: doi: 10.1186/S12913-021-06805-6

8. Gálvez ACM, Sánchez FJ, Moreno CA, Pérez Fernández AJ, García RB, López MC, Ramírez MPB, López MC, Burrero LV, Berja PJ, et al. Value-Based Healthcare in Ostomies. *Int J Environ Res Public Health* (2020) 17:1–15. doi: 10.3390/IJERPH17165879

9. Nielsen VW, Johansen CB, Todberg T, Skov L, Nissen C V., Dodge R, Egeberg A, Thyssen JP, Thomsen SF. A value-based healthcare model for initiating and switching psoriasis therapies-Results from the prospective multicentre IMPROVE study. *J Eur Acad Dermatol Venereol* (2024) 38:844–850. doi: 10.1111/JDV.19690

10. Theunissen L, Cremers HP, Dekker L, Janssen H, Burg M, Huijbers E, Voermans P, Kemps H, Van Veghel D. Implementing Value-Based Health Care Principles in the Full Cycle of Care: The Pragmatic Evolution of the Netherlands Heart Network. *Circ Cardiovasc Qual Outcomes* (2023) 16:E009054. doi: 10.1161/CIRCOUTCOMES.122.009054/ASSET/42EDDB08-7B6C-4E03-8A41-8539D0CC3D13/ASSETS/GRAPHIC/CIRCOUTCOMES.122.009054.FIG01.JPG

11. Van Dam VS, Van Zijl FVWJ, Kremer B, Datema FR. The Rhinoplasty Healthcare Monitor: An update on the Practical and Clinical benefits after 10 years of Prospective Outcome Measurements. *Facial Plastic Surgery* (2023) 40:539–545. doi: 10.1055/A-2218-7189/BIB
